# Relationship Between the Risk of Obstructive Sleep Apnea and Cardiovascular Health in Middle-Aged Korean Men and Women: A Nationwide Study

**DOI:** 10.3390/jcm13226702

**Published:** 2024-11-07

**Authors:** Seo Young Kang, Jung Hwan Kim, Yunmi Kim

**Affiliations:** 1 Department of Family Medicine, Uijeongbu Eulji Medical Center, Eulji University School of Medicine, Uijeongbu-si 11759, Republic of Korea; sykang@eulji.ac.kr; 2 Department of Family Medicine, Gangnam Eulji Medical Center, Seoul 06047, Republic of Korea; 12thrib@hanmail.net; 3 College of Nursing, Eulji University, Seongnam-si 13135, Republic of Korea

**Keywords:** obstructive sleep apnea, cardiovascular health, STOP-Bang

## Abstract

**Background/Objectives:** Cardiovascular health (CVH) can be conceptualized as encompassing seven health behaviors and metabolic factors that contribute to cardiovascular disease. We explored the relationship between the risk of obstructive sleep apnea (OSA) and CVH among middle-aged Korean adults. **Methods:** Data from 5909 participants, aged between 40 and 64 years, in the Korea National Health and Nutrition Examination Survey (2019–2021) were analyzed. The risk of OSA was assessed using STOP-Bang questionnaire. CVH metrics, including smoking status, diet, physical activity, body mass index (BMI), blood pressure, total cholesterol level, and fasting glucose concentration, were evaluated using American Heart Association criteria. Multivariate logistic regression analysis was employed to investigate the association between OSA risk and CVH. **Results:** Among study participants, 78.6% of men and 16.3% of women displayed moderate-to-high risk of OSA, while 45.4% of men and 17.2% of women exhibited poor CVH. The ORs (95% CIs) for poor CVH were 2.69 (2.08–3.49) for men at moderate risk of OSA and 6.54 (4.81–8.90) for those at high risk, compared to men at low risk. For women, the ORs were 3.21 (2.47–4.19) for those with moderate risk and 12.88 (6.29–26.38) for those with high risk of OSA, compared to women at low risk. CVH metrics associated with moderate-to-high OSA risk included high BMI, high blood pressure, elevated fasting glucose, and smoking. **Conclusions:** The risk of OSA was associated with poor CVH, while various CVH components were linked to moderate-to-high OSA risk. Managing both OSA and components of CVH is essential to minimize poor CVH.

## 1. Introduction

Cardiovascular diseases (CVDs) are the leading cause of death worldwide, and their prevalence is on the rise [1]. From 1990 to 2019, the global prevalence of CVDs nearly doubled, accompanied by an increase in cardiovascular-related deaths and higher disease burden as measured by disability-adjusted life years [1]. The incidence of CVDs is particularly high among older adults, as these conditions often result from the cumulative effects of poor lifestyle choices and inadequately managed cardiometabolic profiles [2]. Consequently, it is crucial to adopt a healthy lifestyle and maintain an optimal metabolic profile starting at a younger age to prevent the onset of CVDs.

Several key cardiovascular health (CVH) metrics, known as Life’s Simple 7, were developed by the American Heart Association (AHA) to reduce CVD morbidity and mortality. This approach promotes health with respect to seven CVH behaviors and metabolic factors: smoking status, diet, physical activity, body mass index (BMI), blood pressure, total cholesterol level, and fasting glucose concentration [3]. Each CVH metric is evaluated as ideal, intermediate, or poor, depending on the individual’s risk level and treatment status. Previous studies have demonstrated that “ideal” CVH, as assessed by these metrics, is strongly and inversely associated with the risk of CVD morbidity and mortality [4,5,6]. In this context, it is concerning that the prevalence of ideal CVH has declined over a 12-year period among Korean adults [7].

Obstructive sleep apnea (OSA) is characterized by repeated episodes of upper airway obstruction during sleep, resulting in oxygen desaturation and sleep fragmentation [8]. Clinically, common screening for this condition employs the STOP-Bang questionnaire, an instrument comprising eight short items that correspond to the clinical features of OSA [9]. Patients with OSA experience intermittent hypoxia during sleep, which triggers sympathetic activation, inflammation, and oxidative stress, ultimately contributing to cardiometabolic dysfunction [10]. Consequently, OSA is linked to an elevated risk of CVDs [11,12].

The association between OSA and CVD risk has been vigorously investigated previously. In a prospective study in the United States, OSA was associated with an increased risk of incident coronary heart disease and heart failure in men [13]. Another population-based study in Finland showed that OSA increased the risk of coronary heart disease, type 2 diabetes, and diabetic kidney disease [14]. Based on longitudinal studies, several meta-analyses reported an increased risk of CVD and cardiovascular mortality among patients with OSA [11,15,16].

Nevertheless, to our knowledge, no studies have yet evaluated the association between the risk of OSA and overall CVH using CVH metrics developed by the AHA. Given that CVH metrics encompass both behavioral and metabolic factors that contribute to CVDs, exploring the relationship between OSA and CVH could offer valuable insights into how the clinical features of OSA relate to the comprehensive cardiometabolic profiles associated with CVDs. Therefore, this study was conducted to investigate the association between the risk of OSA and CVH in middle-aged Korean adults, utilizing a nationally representative sample.

## 2. Materials and Methods

### 2.1. Study Population

**Figure 1:**
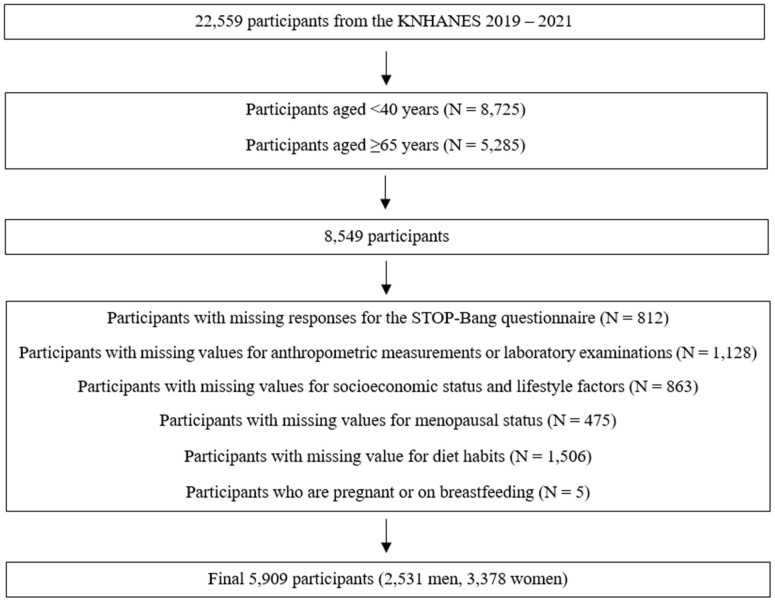
Selection of the study population.

We utilized the data from the eighth Korea National Health and Nutrition Examination Survey (KNHANES) 2019–2021. The KNHANES is a nationwide cross-sectional survey conducted by the Korea Disease Control and Prevention Agency (KDCA). This survey employs a stratified, complex, clustered, and multistage probability sampling method based on participant age, sex, and geographic location. The study design and methodology have been extensively documented in the existing literature [17]. For the KNHANES, trained interviewers meet with participants and complete questionnaires. The data collection for the eighth KNHANES was performed between 2019 and 2021. The institutional review board of the KDCA approved the study protocol, and informed consent was obtained from all participants involved in the KNHANES. The institutional review board of Eulji University provided exempt approval for this study (EUIRB2023-073), and this study was performed in accordance with the Declaration of Helsinki. The inclusion criteria were (1) participants aged between 40 and 64 years and (2) those who completed the STOP-Bang questionnaires. The exclusion criteria were (1) those whose CVH metrics were not assessable due to missing responses, (2) those whose menopausal status was not assessable, and (3) those who were pregnant or breastfeeding. Of the 22559 participants in the eighth KNHANES, we initially selected 8549 respondents aged between 40 and 64 years for the present analysis. This age range was chosen because the STOP-Bang questionnaire, which assesses the risk of OSA, is administered to individuals aged 40 years and older, and the OSA phenotype in older adults differs from that in younger and middle-aged adults (Figure 1) [18]. From the 8549 respondents, we excluded those with missing STOP-Bang questionnaire data (*n* = 812), incomplete anthropometric measurements or laboratory examination data (*n* = 1128), absent information on socioeconomic status or lifestyle factors (*n* = 863), undefined menopausal status (*n* = 475), and unreported dietary habits (*n* = 1506), as well as those who were pregnant or breastfeeding (*n* = 5). Ultimately, the final sample size for our analysis comprised 5909 participants, including 2531 men and 3378 women.

### 2.2. Sociodemographic and Lifestyle Characteristics

Sociodemographic characteristics, including age, sex, area of residence, household income, educational level, occupation, and marital status, were evaluated. Area of residence was categorized as either urban or rural, and household income was divided into quartiles. Educational level was categorized as middle school graduate or less, high school graduate, or college graduate or higher, and occupation was ultimately categorized as no occupation, manual work, or non-manual work. As an initial step, occupation was divided into the following 10 categories according to the Korean Standard Classification of Occupation: managers, professionals, clerks, service workers, sales workers, skilled agriculture/forestry/fishery workers, craft and trade workers, equipment/machine operators and assemblers, those in elementary occupations, and members of the armed forces [19] Managers, professionals, and clerks were categorized as non-manual workers, while the rest were considered manual workers. Unemployed individuals, students, and housewives were classified as having no occupation. Marital status was categorized as either married/cohabitating or unmarried/divorced/separated/widowed.

Lifestyle factors, including alcohol consumption, smoking status, physical activity, and dietary habits, were also assessed. High-risk drinking was defined as consuming at least seven glasses on a single occasion for men and at least five glasses for women [20] Smoking status was categorized as never smoker, former smoker, or current smoker. Physical activity was classified into 3 categories: ideal, intermediate, and poor. Ideal physical activity was achieved when participants engaged in ≥150 min per week of moderate-intensity physical activity, ≥75 min per week of high-intensity physical activity, or a combination of moderate and high-intensity activities totaling ≥150 min per week. Intermediate physical activity was defined as 1–149 min per week of moderate-intensity activity, 1–74 min per week of high-intensity activity, or a combination of both totaling 1–149 min per week. Poor physical activity status was assigned to participants who did not meet the criteria for the above categories [21] Dietary habits were evaluated using the 24 h recall method, with assessments of the daily intake of total calories, carbohydrates, protein, fat, sodium, and fiber. Additionally, menopausal status was evaluated for women.

### 2.3. Anthropometric and Laboratory Variables

Anthropometric variables were measured using standardized techniques and equipment, with participants dressed in a lightweight gown. Height was determined to the nearest 0.1 cm with a portable anthropometer, with participants standing erect, and weight was measured to the nearest 0.1 kg using a balanced scale. BMI (in kg/m^2^) was calculated by dividing the weight in kilograms by the square of the height in meters. Neck circumference was measured to the nearest 0.1 cm; participants were seated with a right-angle posture during the measurement. Neck circumference was measured at the position directly under the Adam’s apple for men and directly below the thyroid cartilage for women. This measurement was performed twice, and the average of these values was used for the subsequent analysis. Systolic blood pressure (SBP) and diastolic blood pressure (DBP) were measured with a sphygmomanometer while participants were seated. These measurements were taken 3 times at 5 min intervals, and the mean of the second and third measurements was used. Blood samples were collected following a minimum 8 h fast, and biochemical values were analyzed in a certified laboratory using a Hitachi Automatic Analyzer 7600 (Hitachi, Tokyo, Japan). Levels of fasting glucose and total cholesterol, both reported in milligrams per deciliter (mg/dL), were also measured.

### 2.4. Classification of OSA Risk

We assessed the risk of OSA using the STOP-Bang questionnaire. This instrument comprises 8 items, with the total number of affirmative (“yes”) responses to these items used to gauge the risk of OSA. The items include (1) snoring, as determined by the question “Do you snore loudly?”; (2) tiredness, assessed with “Do you often feel tired, fatigued, or sleepy during the day?”; (3) observed apnea, determined by “Has anyone observed you stop breathing during your sleep?”; (4) high blood pressure, defined as SBP ≥ 140 mmHg, DBP ≥ 90 mmHg, or the use of antihypertensive medication; (5) BMI greater than 30 kg/m^2^; (6) age above 50 years; (7) neck circumference above 36.3 cm; and (8) male sex [22]. The risk levels for OSA were categorized as high, intermediate, and low risk, corresponding to total “yes” responses of 5–8, 3–4, and 0–2, respectively.

### 2.5. Definitions of CVH

CVH metrics were assessed using criteria established by the AHA [7,21,23,24]. These metrics include smoking status, diet, physical activity, BMI, blood pressure, total cholesterol level, and fasting glucose concentration. Each CVH metric was assigned a score of 2, 1, or 0, corresponding to ideal, intermediate, or poor status, respectively. For the dietary component, we adhered to the Dietary Approaches to Stop Hypertension and the Korean dietary guidelines for dyslipidemia [21]. A score of 1 was allocated to each element of a healthy diet, which includes fat intake of less than 35% of total daily energy intake, protein intake of more than 15% of total daily energy intake, carbohydrate intake of less than 55% of total daily energy intake, sodium intake of less than 2300 mg/day, and fiber intake of more than 20 g/day. The scores on these 5 dietary components were summed to obtain the overall diet metric. For the BMI component, we applied the World Health Organization recommendations for Asian populations, as has been applied in previous studies [7,21].

The criteria for ideal, intermediate, and poor CVH were defined for each metric as follows: (1) smoking status: never smoker (ideal), former smoker (intermediate), and current smoker (poor); (2) diet: a score of 4–5 (ideal), 2–3 (intermediate), and 0–1 (poor); (3) physical activity as previously defined; (4) BMI less than 23 kg/m^2^ (ideal), 23–24.9 kg/m^2^ (intermediate), and 25 kg/m^2^ or higher (poor); (5) blood pressure: SBP less than 120 mmHg and DBP less than 80 mmHg (ideal), SBP 120–139 mmHg or DBP 80–89 mmHg or treated to goal (intermediate), and SBP 140 mmHg or higher or DBP 90 mmHg or higher (poor); (6) total cholesterol less than 200 mg/dL (ideal), 200–239 mg/dL or treated to goal (intermediate), and 240 mg/dL or higher (poor); and (7) fasting glucose less than 100 mg/dL (ideal), 100–125 mg/dL or treated to goal (intermediate), and 126 mg/dL or higher (poor). The overall CVH metric was assessed by summing the scores of each CVH metric. A total score of 12 or higher was classified as ideal, 8–11 as intermediate, and 7 or lower as poor CVH.

### 2.6. Statistical Analysis

All analyses were performed separately for men and women, and we performed normality tests to evaluate the distribution of data. Descriptive statistics were used to represent the basic characteristics of the study participants categorized by CVH status. The chi-square test and 1-way analysis of variance were utilized to compare participant characteristics across categorical and continuous variables, respectively. For continuous variables, a post hoc analysis using the Tukey test was conducted. The chi-square test was also used to compare the components of the STOP-Bang score by CVH status. To identify factors associated with poor CVH, we performed multivariate logistic regression analysis. We calculated the odds ratios (ORs) and 95% confidence intervals (CIs) for poor CVH, comparing those at moderate and high risk of OSA to those at low risk. In this analysis, the independent variables were age, area of residence, household income, educational level, occupation, marital status, high-risk drinking, menopausal status, as well as OSA risk; whereas, the dependent variable was CVH status. Additionally, we assessed the ORs and 95% CIs for moderate-to-high risk of OSA in each CVH metric component using multivariate logistic regression analysis. In this analysis, the independent variables were age and each CVH component, whereas the dependent variable was OSA risk. The analyses were performed using SAS version 9.4 (SAS Institute, Cary, NC, USA). A 2-tailed *p*-value of less than 0.05 was considered to indicate statistical significance.

## 3. Results

### 3.1. Basic Participant Characteristics by CVH Status

Table 1 presents the basic characteristics of the study participants categorized by CVH status. Among women, higher proportions of poor CVH were observed among older age groups, lower income brackets, manual laborers, those who were not married or cohabitating, and postmenopausal participants (*p* < 0.05). In contrast, no significant association was found between poor CVH and age, income, occupation, or marital status in men. The rate of poor CVH was greater among residents of rural areas and individuals with a lower educational level for both men and women (*p* < 0.05). For men, a higher proportion of participants with poor CVH was noted among high-risk drinkers (*p* < 0.001), whereas no significant association was observed among women. BMI, SBP, DBP, fasting glucose concentration, and total cholesterol level all increased with the deterioration of CVH in both men and women (*p* < 0.001).

### 3.2. STOP-Bang Scores and CVH

**Figure 2:**
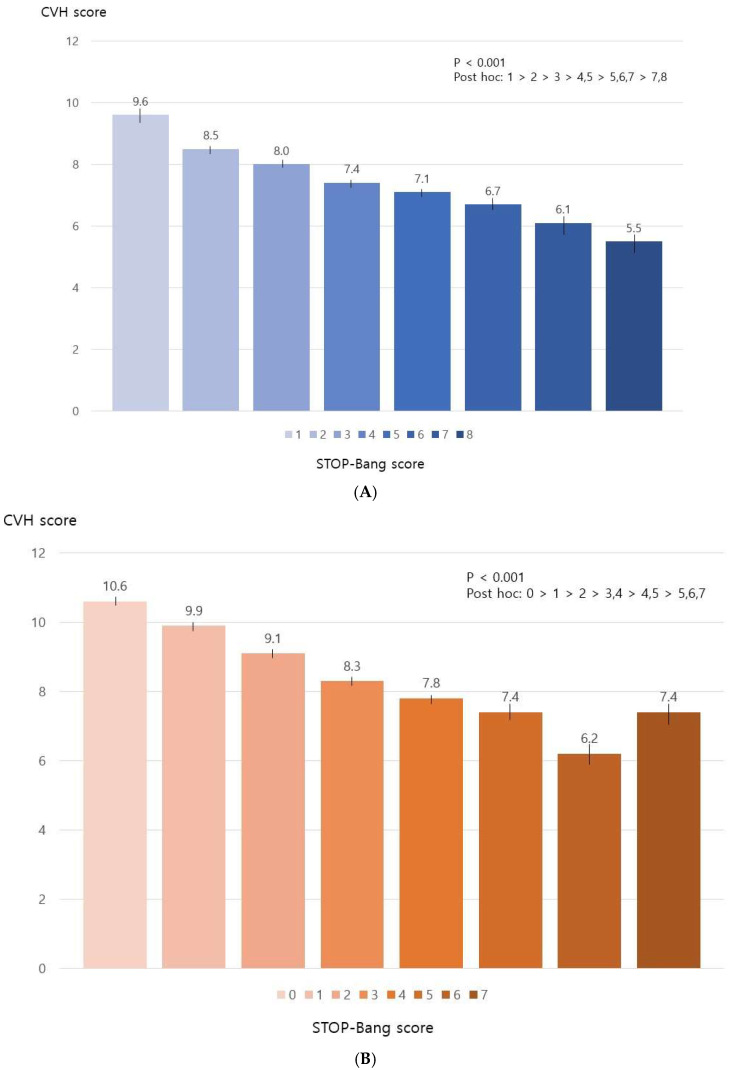
Relationship between STOP-Bang score and CVH score in Korean middle-aged Korean men and women. (**A**) Men; (**B**) Women.

Figure 2 illustrates the mean CVH scores of the study participants in relation to their STOP-Bang scores. The mean CVH score tended to decline with increasing STOP-Bang score (*p* < 0.001). Table 2 presents the distribution of CVH status across the individual components of the STOP-Bang score. For both men and women, higher proportions of poor CVH were observed among participants who snored, who reported apnea being witnessed during sleep, who exhibited high blood pressure, who had a BMI greater than 30 kg/m^2^, and who displayed a neck circumference exceeding 36.3 cm. Furthermore, poor CVH was found in greater proportions among men who reported experiencing daytime tiredness, fatigue, or sleepiness and among women over 50 years old. Lastly, a greater percentage of participants with a higher risk for OSA also exhibited poor CVH (*p* < 0.05).

### 3.3. Factors Associated with Poor CVH

Table 3 presents the results of the multivariate analysis, which was conducted to identify factors associated with poor CVH among the study participants. The findings revealed that in men, the odds of poor CVH decreased with advancing age, while in both men and women, it increased as educational level declined. For women, the odds of poor CVH were greater among manual workers (OR, 1.36; 95% CI, 1.05–1.74; *p* = 0.018) compared to those with no occupation. In men, the odds of poor CVH were relatively high among those not married or cohabitating (OR, 1.31; 95% CI, 1.02–1.67; *p* = 0.033). Additionally, individuals identified as high-risk drinkers displayed higher odds of poor CVH compared to non-drinkers. The odds of poor CVH also escalated with increasing risk of OSA. Specifically, men with moderate and high risk of OSA exhibited odds ratios of 2.69 (95% CI, 2.08–3.49; *p* < 0.001) and 6.54 (95% CI, 4.81–8.90; *p* < 0.001), respectively, while women with moderate and high risk of OSA had odds ratios of 3.21 (95% CI, 2.47–4.19; *p* < 0.001) and 12.88 (95% CI, 6.29–26.38; *p* < 0.001), respectively, when compared to those with a low risk of OSA.

### 3.4. Association Between Moderate-to-High Risk of OSA and CVH Metrics

Table 4 displays the relationship between each CVH metric and moderate-to-high risk of OSA. In the multivariate model, the odds of moderate-to-high OSA risk increased with advancing age, higher BMI, and worsening blood pressure in both men and women. Relative to never-smokers, the odds of moderate-to-high risk of OSA were higher among former smokers (OR 1.56; 95% CI, 1.11–2.20; *p* = 0.011) in men and current smokers (OR 2.50; 95% CI, 1.43–4.36) in women. Men with a total cholesterol level of 200–240 mg/dL or those who had been treated to goal displayed increased odds (1.34; 95% CI, 1.03–1.74; *p* = 0.030) of moderate-to-high OSA risk compared to men with a total cholesterol level below 200 mg/dL without treatment. Additionally, the odds were higher for men with a fasting glucose level of 126 mg/dL or above (2.11; 95% CI, 1.03–4.30; *p* = 0.040) and for women with a fasting glucose level of 100–126 mg/dL or treated to the goal (1.84; 95% CI 1.41–2.38; *p* < 0.001), as opposed to men and women with fasting glucose levels below 100 mg/dL without treatment.

## 4. Discussion

In this study, the risk of OSA was associated with increased odds of poor CVH. The likelihood of poor CVH escalated as the risk of OSA increased, with a pronounced effect observed in women than men. Additional factors linked to poor CVH included lower educational level, high-risk drinking, not being married or cohabitating (among men), and lower household income and manual occupation (among women). Notably, the odds of poor CVH decreased with advancing age in men. The CVH metrics that were associated with a moderate-to-high risk of OSA were elevated BMI, increased blood pressure, elevated fasting glucose level, and smoking.

The relationship between OSA and poor CVH may be explained by several underlying mechanisms. The primary pathophysiological features of OSA include hypoxemia, autonomic dysfunction, sleep disruption, intrathoracic pressure changes, and hypercapnia [10,25]. Notably, intermittent hypoxemia, a defining characteristic of OSA, mediates cardiovascular complications through various pathways such as inflammation, metabolic dysregulation, sympathetic nervous system activation, and oxidative stress [10,25]. The recurrent arousals caused by sleep disruption, intrathoracic pressure variations, and concomitant obesity further contribute to these adverse effects [10,25]. Consequently, the AHA has recommended OSA screening for patients with resistant or poorly controlled hypertension, pulmonary hypertension, or recurrent atrial fibrillation, and it has advised considering a sleep study for patients exhibiting New York Heart Association class II–IV heart failure symptoms, tachy-brady syndrome, sick sinus syndrome, and ventricular tachycardia, along with survivors of sudden cardiac arrest and those with a history of stroke [26].

Other factors associated with poor CVH in this study included a lower educational level, high-risk drinking, and (among men) not being married or cohabitating. Among women, lower household income and manual occupation were linked to poor CVH. Low socioeconomic status is an established risk factor for poor cardiovascular outcomes [27]. Regarding employment status, while unemployment has been associated with an increased risk for CVDs in previous studies [28,29], this study indicated that being a manual worker was associated with even higher odds of poor CVH among Korean women. Therefore, sustainable and effective interventions to improve CVH should be particularly emphasized among populations with low socioeconomic status. Traditionally, being unmarried, divorced, or widowed has been associated with an increased risk for CVDs [30]. In this study, however, this association was only observed among men, reflecting gender norms and the role of women in providing care to their families in Korea. Men with spouses are likely to receive healthcare support from their partners, whereas women with spouses generally do not receive the same level of care. High-risk drinking is known to increase the risk of CVDs, as alcohol modulates the pathophysiological processes of inflammation and atherosclerosis [31]. Alcohol consumption can modify CVD risk factors by altering lipid profiles, carotid intima-media thickness, and insulin sensitivity. It also influences hemostatic factors such as platelet reactivity and fibrinogen levels and modulates ischemia-reperfusion mechanisms [31].

In this study, we observed that the odds of poor CVH decreased among older male age groups, despite the general trend of increasing CVD risk with advancing age [1]. Here, CVD refers to metrics that include both behavioral patterns and metabolic factors. Therefore, the higher odds of poor CVH observed in Korean men in their 40s suggest that these individuals are less likely to engage in healthy lifestyle behaviors and to have favorable metabolic profiles compared to their older counterparts. Notably, the prevalence of obesity, diabetes, and hypercholesterolemia is rapidly rising among men in their 40s in Korea [32,33]. It is crucial to emphasize the maintenance of healthy lifestyles and the management of cardiovascular risk factors from a younger age to prevent CVD onset in Korean men.

Although the prevalence of OSA is generally higher in men than in women [34], this study indicated that the likelihood of poor CVH was greater in women at high risk for OSA compared to their male counterparts. Woodward identified three distinct ways in which women are at a disadvantage regarding CVDs [35]. As prior research has indicated, women are less likely to receive both personal and professional health care compared to men, and the impact of cardiovascular risk factors on the development of CVDs manifests differently between sexes [35]. For example, common cardiovascular risk factors such as smoking, diabetes, atrial fibrillation, and low socioeconomic status have been shown to exert a stronger relative effect on CVDs in women than in men [36,37,38,39]. This phenomenon may extend to the sex difference observed in the association between the risk of OSA and poor CVH, although the underlying mechanisms remain unclear. Notably, screening and evaluation for CVH must not be overlooked in women at risk of OSA.

The components of CVH associated with moderate-to-high risk of OSA identified in this study include high BMI, high blood pressure, elevated fasting glucose level, and smoking. These findings align with risk factors reported in prior research [8]. The metabolic conditions that are considered risk factors for OSA may also be exacerbated by OSA itself, as intermittent hypoxemia is known to mediate cardiovascular complications [10,25] Given the potential for a vicious cycle due to the bidirectional relationship between OSA and cardiometabolic comorbidities, it is necessary to implement simultaneous interventions targeting both OSA and CVH components to achieve good CVH [40].

This study has several limitations. First, we assessed the risk of OSA using the STOP-Bang questionnaire instead of diagnostic criteria, as the KNHANES database includes only STOP-Bang questionnaire results. Differences may be observed when applying the diagnostic criteria, although the STOP-Bang questionnaire has demonstrated high validity for OSA screening [41] Since OSA is a complex entity that must be documented by polysomnography, our assessment for OSA may not encompass the comprehensive nature of OSA. Second, recall bias could have affected the classifications of variables in this study, as most lifestyle variables were determined by participant responses to survey questions. Lastly, the cross-sectional design of our study precludes the establishment of a causal relationship between OSA risk and CVH. However, the association between OSA and CVH is noteworthy, given their bidirectional relationship. Our study highlights the associations between OSA and comprehensive cardiometabolic profiles that contribute to CVDs.

## 5. Conclusions

The risk of OSA was associated with poor CVH in middle-aged Korean men and women. Additionally, several CVH components, including high BMI, high blood pressure, elevated fasting glucose level, and smoking, were linked to a moderate-to-high risk of OSA. The effective management of both OSA and components of CVH is necessary to minimize poor CVH.

## Figures and Tables

**Table 1:** Basic characteristics of the study participants by CVH status.

	Men (*n* = 2531)				Women (*n* = 3378)			
	Poor CVH (*n* = 1149)	Intermediate CVH (*n* = 1312)	Ideal CVH (*n* = 70)	*p*-Value	Poor CVH (*n* = 580)	Intermediate CVH (*n* = 2206)	Ideal CVH (*n* = 592)	*p*-Value
Age group (years)								
40–44	213 (45.0)	248 (52.4)	12 (2.5)	0.205	56 (9.0)	386 (62.0)	181 (29.1)	<0.001
45–49	212 (43.0)	270 (54.8)	11 (2.2)		81 (11.9)	437 (64.0)	165 (24.2)	
50–54	226 (50.3)	211 (47.0)	12 (2.7)		125 (19.1)	439 (67.2)	89 (13.6)	
55–59	238 (44.1)	281 (52.0)	21 (3.9)		141 (20.0)	471 (66.8)	93 (13.2)	
60–64	260 (45.1)	302 (52.4)	14 (2.4)		177 (24.8)	473 (66.2)	64 (9.0)	
Are of residence								
Urban	887 (44.0)	1073 (53.3)	54 (2.7)	0.004	439 (15.9)	1809 (65.4)	516 (18.7)	0.003
Rural	262 (50.7)	239 (46.2)	16 (3.1)		141 (23.0)	397 (64.7)	76 (12.4)	
Household income								
First quartile (low)	127 (52.5)	108 (44.6)	7 (2.9)	0.082	85 (27.3)	195 (62.7)	31 (10.0)	<0.001
Second quartile	246 (46.2)	273 (51.2)	14 (2.6)		154 (18.7)	538 (65.5)	130 (15.8)	
Third quartile	360 (46.1)	404 (51.7)	17 (2.2)		165 (16.4)	671 (66.6)	172 (17.1)	
Fourth quartile (high)	416 (42.7)	527 (54.1)	32 (3.3)		176 (14.2)	802 (64.8)	259 (20.9)	
Educational level								
<Middle school graduate	195 (54.0)	161 (44.6)	5 (1.4)	<0.001	188 (29.5)	402 (63.1)	47 (7.4)	<0.001
High school graduate	444 (48.3)	457 (49.7)	19 (2.1)		253 (18.3)	913 (65.9)	219 (15.8)	
≥College graduate	510 (40.8)	694 (55.5)	46 (3.7)		139 (10.3)	891 (65.7)	326 (24.0)	
Occupation								
None	183 (46.3)	199 (50.4)	13 (3.3)	0.069	222 (16.3)	898 (66.0)	241 (17.7)	<0.001
Manual work	594 (47.1)	637 (50.6)	29 (2.3)		254 (21.8)	747 (64.2)	163 (14.0)	
Non-manual work	372 (42.5)	476 (54.3)	28 (3.2)		104 (12.2)	561 (65.8)	188 (22.0)	
Marital status								
Married/cohabitating	916 (44.2)	1097 (52.9)	60 (2.9)	0.088	457 (16.4)	1817 (65.2)	511 (18.3)	0.002
Unmarried/divorced/separated/widowed	233 (50.9)	215 (46.9)	10 (2.2)		123 (20.7)	389 (65.6)	81 (13.7)	
High risk drinking								
Non-drinker	329 (38.8)	477 (56.2)	43 (5.1)	<0.001	379 (16.3)	1539 (66.3)	405 (17.4)	0.147
<1/month	176 (43.8)	218 (54.2)	8 (2.0)		77 (15.9)	311 (64.3)	96 (19.8)	
≥1/month	644 (50.3)	617 (48.2)	19 (1.5)		124 (21.7)	356 (62.3)	91 (15.9)	
Menopausal status								
Premenopausal	N/A	N/A	N/A	N/A	176 (11.6)	974 (64.0)	373 (24.5)	<0.001
Postmenopausal	N/A	N/A	N/A		404 (21.8)	1232 (66.4)	219 (11.8)	
BMI (kg/m^2^)	26.5 (0.1)	23.7 (0.1)	21.6 (0.2)	<0.001	27.2 (0.2)	23.4 (0.1)	21.2 (0.1)	<0.001
SBP (mmHg)	126.0 (0.5)	117.7 (0.5)	107.5 (1.3)	<0.001	126.5 (0.8)	115.9 (0.4)	106.1 (0.5)	<0.001
DBP (mmHg)	83.1 (0.3)	77.8 (0.2)	72.5 (0.8)	<0.001	79.8 (0.4)	75.3 (0.2)	69.6 (0.3)	<0.001
Fasting glucose (mg/dL)	115.2 (1.1)	100.7 (0.5)	93.7 (0.9)	<0.001	115.4 (1.7)	97.9 (0.5)	90.6 (0.3)	<0.001
Total cholesterol (mg/dL)	202.8 (1.4)	190.0 (1.0)	181.2 (3.7)	<0.001	211.7 (2.3)	202.2 (0.9)	185.1 (1.2)	<0.001

CVH: cardiovascular health, BMI: body mass index, SBP: systolic blood pressure, DBP: diastolic blood pressure. Values are presented as unweighted number (weighted percentage) or mean (standard error).

**Table 2:** CVH status according to each component of the STOP-Bang score in middle-aged Korean men and women.

	Men (*n* = 2531)				Women (*n* = 3378)			
	Poor CVH (*n* = 1149)	Intermediate CVH (*n* = 1312)	Ideal CVH (*n* = 70)	*p*-Value	Poor CVH (*n* = 580)	Intermediate CVH (*n* = 2206)	Ideal CVH (*n* = 592)	*p*-Value
Snoring								
No	747 (42.0)	970 (54.6)	61 (3.4)	<0.001	448 (15.3)	1934 (65.9)	554 (18.9)	<0.001
Yes	402 (53.4)	342 (45.4)	9 (1.2)		132 (29.9)	272 (61.5)	38 (8.6)	
Daytime tiredness, fatigue, or sleepiness								
No	778 (43.6)	953 (53.4)	55 (3.1)	0.009	375 (16.5)	1490 (65.7)	403 (17.8)	0.364
Yes	371 (49.8)	359 (48.2)	15 (2.0)		205 (18.5)	716 (64.5)	189 (17.0)	
Observed cessation of breathing during sleep								
No	892 (43.4)	1102 (53.7)	59 (2.9)	<0.001	545 (16.8)	2131 (65.6)	574 (17.7)	0.007
Yes	257 (53.8)	210 (43.9)	11 (2.3)		35 (27.3)	75 (58.6)	18 (14.1)	
High blood pressure								
No	567 (34.8)	998 (61.3)	64 (3.9)	<0.001	286 (11.1)	1718 (66.7)	570 (22.1)	<0.001
Yes	582 (64.5)	314 (34.8)	6 (0.7)		294 (36.6)	488 (60.7)	22 (2.7)	
BMI (kg/m^2^)								
≤30	1012 (42.9)	1279 (54.2)	70 (3.0)	<0.001	489 (15.3)	2110 (66.1)	592 (18.6)	<0.001
>30	137 (80.6)	33 (19.4)	0 (0.0)		91 (48.7)	96 (51.3)	0 (0.0)	
Age (years)								
≤50	475 (45.2)	550 (52.4)	25 (2.4)	0.585	155 (10.8)	902 (63.0)	374 (26.1)	<0.001
>50	674 (45.5)	762 (51.5)	45 (3.0)		425 (21.8)	1304 (67.0)	218 (11.2)	
Neck circumference (cm)								
≤36.3	92 (18.2)	371 (73.5)	42 (8.3)	<0.001	489 (15.2)	2129 (66.3)	592 (18.4)	<0.001
>36.3	1057 (52.2)	941 (46.4)	28 (1.4)		91 (54.2)	77 (45.8)	0 (0.0)	
Risk of obstructive sleep apnea								
Low (0–2)	135 (24.9)	372 (68.6)	35 (6.5)	<0.001	372 (13.1)	1885 (66.6)	572 (20.2)	<0.001
Moderate (3–4)	616 (44.8)	729 (53.0)	31 (2.3)		176 (35.2)	306 (61.2)	18 (3.6)	
High (5–8)	398 (64.9)	211 (34.4)	4 (0.7)		32 (65.3)	15 (30.6)	2 (4.1)	

CVH: cardiovascular health, BMI: body mass index.

**Table 3:** Multivariate analysis for factors associated with poor CVH in middle-aged Korean men and women.

	Men				Women			
	Crude OR (95% CI)	*p*	* Adjusted OR (95% CI)		Crude OR (95% CI)		^†^ Adjusted OR (95% CI)	
Age group (years)								
40–44	1.00		1.00		1.00		1.00	
45–49	0.96 (0.73–1.26)	0.747	0.92 (0.69–1.23)	0.584	1.17 (0.74–1.84)	0.510	1.04 (0.66–1.64)	0.875
50–54	1.29 (0.96–1.73)	0.093	0.84 (0.61–1.17)	0.307	2.07 (1.35–3.18)	0.001	1.38 (0.83–2.30)	0.218
55–59	1.02 (0.77–1.36)	0.875	0.62 (0.46–0.84)	0.002	2.19 (1.48–3.22)	<0.001	1.11 (0.63–1.95)	0.718
60–64	1.03 (0.79–1.35)	0.839	0.58 (0.42–0.80)	0.001	2.83 (1.93–4.15)	<0.001	1.16 (0.64–2.13)	0.624
Area of residence								
Urban	1.00		1.00		1.00		1.00	
Rural	1.40 (1.11–1.75)	0.004	1.26 (1.00–1.59)	0.054	1.56 (1.17–2.07)	0.003	1.25 (0.92–1.70)	0.158
Household income								
Fourth quartile	1.00		1.00		1.00		1.00	
Third quartile	1.13 (0.91–1.41)	0.264	1.11 (0.87–1.42)	0.388	1.17 (0.90–1.53)	0.239	1.01 (0.77–1.32)	0.965
Second quartile	1.13 (0.88–1.45)	0.337	1.03 (0.78–1.36)	0.852	1.62 (1.24–2.12)	0.001	1.25 (0.92–1.70)	0.152
First quartile	1.60 (1.15–2.23)	0.006	1.37 (0.92–2.05)	0.118	2.38 (1.69–3.37)	<0.001	1.57 (1.05–2.36)	0.029
Educational level								
≥College graduate	1.00		1.00		1.00		1.00	
High school graduate	1.33 (1.10–1.61)	0.004	1.29 (1.02–1.62)	0.033	2.00 (1.55–2.57)	<0.001	1.57 (1.18–2.11)	0.002
<Middle school graduate	1.76 (1.34–2.31)	<0.001	1.93 (1.38–2.71)	<0.001	3.93 (2.94–5.25)	<0.001	2.20 (1.48–3.26)	<0.001
Occupation								
None	1.00		1.00		1.00		1.00	
Manual work	0.99 (0.75–1.30)	0.934	1.05 (0.77–1.43)	0.768	1.50 (1.19–1.90)	0.001	1.36 (1.05–1.74)	0.018
Non-manual work	0.87 (0.66–1.15)	0.315	1.18 (0.83–1.66)	0.355	0.70 (0.53–0.93)	0.013	1.14 (0.82–1.60)	0.429
Marital status								
Married/cohabitating	1.00		1.00		1.00		1.00	
Unmarried/divorced/separated/widowed	1.31 (1.05–1.63)	0.017	1.31 (1.02–1.67)	0.033	1.27 (0.98–1.63)	0.067	0.96 (0.71–1.29)	0.772
High risk drinking								
Non-drinker	1.00		1.00		1.00		1.00	
<1/month	1.25 (0.94–1.65)	0.125	1.40 (1.04–1.89)	0.026	1.06 (0.77–1.46)	0.707	1.14 (0.81–1.60)	0.460
≥1/month	1.58 (1.28–1.95)	<0.001	1.56 (1.25–1.95)	<0.001	1.42 (1.09–1.86)	0.011	1.43 (1.07–1.92)	0.016
Menopausal status								
Premenopausal	N/A		N/A		1.00		1.00	
Postmenopausal					1.94 (1.57–2.41)	<0.001	1.13 (0.74–1.73)	0.574
Risk of obstructive sleep apnea								
Low (0–2)			1.00				1.00	
Moderate (3–4)			2.69 (2.08–3.49)	<0.001			3.21 (2.47–4.19)	<0.001
High (5–8)			6.54 (4.81–8.90)	<0.001			12.88 (6.29–26.38)	<0.001

CVH: cardiovascular health, OR: odds ratio, CI: confidence interval, * Adjusted for age, area of residence, household income, educational level, occupation, marital status, high-risk drinking, and risk of obstructive sleep apnea. ^†^ Adjusted for age, area of residence, household income, educational level, occupation, marital status, high-risk drinking, menopausal status, and risk of obstructive sleep apnea.

**Table 4:** Odds ratios and 95% confidence intervals for moderate-to-high risk of obstructive sleep apnea according to the components of CVH.

		Men		Women				
	Crude OR (95% CI)		* Adjusted OR (95% CI)		Crude OR (95% CI)		* Adjusted OR (95% CI)	
Age group (years)								
40–44	1.00		1.00		1.00		1.00	
45–49	0.94 (0.72–1.23)	0.657	0.91 (0.66–1.26)	0.567	1.42 (0.84–2.390	0.189	1.10 (0.63–1.93)	0.727
50–54	4.62 (3.18–6.72)	<0.001	5.88 (3.80–9.08)	<0.001	4.50 (2.87–7.07)	<0.001	3.40 (2.16–5.37)	<0.001
55–59	5.20 (3.62–7.47)	<0.001	7.95 (5.29–11.95)	<0.001	5.75 (3.68–9.00)	<0.001	4.16 (2.61–6.63)	<0.001
60–64	5.33 (3.59–7.92)	<0.001	7.32 (4.76–11.26)	<0.001	8.25 (5.48–12.44)	<0.001	5.07 (3.32–7.75)	<0.001
Smoking status								
Never smoker	1.00		1.00		1.00		1.00	
Former smoker	1.78 (1.34–2.35)	<0.001	1.56 (1.11–2.20)	0.011	1.07 (0.68–1.68)	0.778	1.40 (0.82–2.41)	0.217
Current smoker	1.16 (0.87–1.54)	0.315	1.09 (0.77–1.53)	0.622	1.70 (1.08–2.66)	0.021	2.50 (1.43–4.36)	0.001
Diet								
Ideal	1.00		1.00		1.00		1.00	
Intermediate	0.71 (0.50–1.02)	0.060	0.72 (0.49–1.06)	0.095	1.19 (0.89–1.59)	0.232	0.91 (0.65–1.28)	0.592
Poor	0.77 (0.46–1.27)	0.301	0.82 (0.44–1.54)	0.536	1.18 (0.73–1.92)	0.506	1.17 (0.66–2.10)	0.591
Physical activity								
Ideal	1.00		1.00		1.00		1.00	
Intermediate	0.84 (0.67–1.05)	0.123	0.72 (0.49–1.06)	0.298	1.07 (0.83–1.39)	0.592	1.11 (0.83–1.48)	0.472
Poor	0.97 (0.76–1.23)	0.784	0.82 (0.44–1.54)	0.274	1.18 (0.92–1.50)	0.187	1.01 (0.76–1.33)	0.970
BMI (kg/m^2^)								
<23	1.00		1.00		1.00		1.00	
23–24.9	2.47 (1.87–3.24)	<0.001	3.22 (2.29–4.53)	<0.001	1.64 (1.19–2.25)	0.003	1.22 (0.86–1.73)	0.259
≥25	5.05 (3.91–6.53)	<0.001	6.38 (4.63–8.78)	<0.001	5.85 (4.53–7.56)	<0.001	3.69 (2.78–4.89)	<0.001
Blood pressure								
Ideal	1.00		1.00		1.00		1.00	
Intermediate	3.89 (3.11–4.88)	<0.001	2.63 (2.03–3.39)	<0.001	8.34 (6.15–11.33)	<0.001	5.07 (3.66–7.03)	<0.001
Poor	16.98 (5.68–50.76)	<0.001	15.57 (5.23–46.40)	<0.001	12.06 (7.21–20.18)	<0.001	8.96 (5.34–15.03)	<0.001
Total cholesterol								
<200 mg/dL (untreated)	1.00		1.00		1.00		1.00	
200–239 mg/dL or treated to goal	1.47 (1.18–1.83)	0.001	1.34 (1.03–1.74)	0.030	1.92 (1.51–2.45)	<0.001	1.10 (0.83–1.46)	0.496
≥240 mg/dL	1.21 (0.86–1.69)	0.273	1.13 (0.74–1.73)	0.562	1.40 (0.99–1.99)	0.058	0.80 (0.54–1.18)	0.265
Fasting glucose								
<100 mg/dL (untreated)	1.00		1.00		1.00		1.00	
100–125 mg/dL or treated to goal	2.03 (1.62–2.55)	<0.001	1.30 (1.00–1.70)	0.053	3.31 (2.65–4.13)	<0.001	1.84 (1.41–2.38)	<0.001
≥126 mg/dL	4.84 (2.55–9.20)	<0.001	2.11 (1.03–4.30)	0.040	4.45 (2.48–7.99)	<0.001	1.58 (0.80–3.12)	0.187

CVH: cardiovascular health, OR: odds ratio, CI: confidence interval, BMI: body mass index. * Adjusted for age, smoking status, diet, physical activity, BMI, blood pressure, total cholesterol, and fasting glucose.

## Data Availability

The data presented in this study are openly available on the website of the Korea Centers for Disease Control and Prevention at https://knhanes.kdca.go.kr/knhanes/sub03/sub03_02_05.do (accessed on 1 August 2023).
